# Evaluation of MALDI-TOF mass spectrometry and MALDI BioTyper in comparison to 16S rDNA sequencing for the identification of bacteria isolated from Arctic sea water

**DOI:** 10.1371/journal.pone.0181860

**Published:** 2017-07-24

**Authors:** Anna Maria Timperio, Susanna Gorrasi, Lello Zolla, Massimiliano Fenice

**Affiliations:** 1 Dipartimento di Ecologia e Biologia, University of Tuscia, Viterbo, Italy; 2 Dipartimento di Scienze Agrarie e Forestali, University of Tuscia, Viterbo, Italy; 3 Laboratorio di Microbiologia Marina Applicata, CONISMA, University of Tuscia, Viterbo, Italy; Pacific Northwest National Laboratory, UNITED STATES

## Abstract

MALDI-TOF Mass Spectrometry in association with the MALDI BioTyper 3.1 software has been evaluated for the identification and classification of 45 Arctic bacteria isolated from Kandalaksha Bay (White Sea, Russia). The high reliability of this method has been already demonstrated, in clinical microbiology, by a number of studies showing high attribution concordance with other credited analyses. Recently, it has been employed also in other branches of microbiology with controversial performance. The phyloproteomic results reported in this study were validated with those obtained by the “gold standard” 16S rDNA analysis. Concordance between the two methods was 100% at the genus level, while at the species level it was 48%. These percentages appeared to be quite high compared with other studies regarding environmental bacteria. However, the performance of MALDI BioTyper changed in relation to the taxonomical group analyzed, reflecting known identification problems related to certain genera. In our case, attribution concordance for *Pseudomonas* species was rather low (29%), confirming the problematic taxonomy of this genus, whereas that of strains from other genera was quite high (> 60%). Among the isolates tested in this study, two strains (*Exiguobacterium oxidotolerans* and *Pseudomonas costantinii*) were misidentified by MALDI BioTyper due to absence of reference spectra in the database. Accordingly, missing spectra were acquired for the database implementation.

## Introduction

Although culture-independent methods provide a wider outlook on microbial diversity and ecology, cultivation of new isolates is still important and routinely carried out in most laboratories of microbiology. This is particularly true for environmental microorganisms, which represent an immense resource of new natural products for food and feed additives, drug development, and other industrial products [1]. Cultivation is also fundamental for a number of institutions dealing with culture collections of microorganisms. In all these cases, pure cultures are needed.

In order to identify and classify new isolates at the species level, various methods are available ranging from simple phenotypic tests, to more complex biochemical assays, up to advanced molecular techniques. Although rather rapid, most phenotypic tests do not supply sufficient and dependable information, since they might consider as discriminatory features some trait dissimilarities, which do not correspond to real species differences [2]. Various genotypic methodologies have been developed to distinguish individual genomic species, including amplification of 16S ribosomal DNA restriction analysis (ARDRA), sequencing of 16S rDNA, tRNA spacer fingerprinting and selective amplification of restriction fragments (AFLP) and DNA-DNA hybridization [3]. Among PCR-based procedures, 16S rDNA sequencing is one of the most used method, at least as first identification attempt, and it is widely considered as the “gold standard” to resolve doubtful cases arising when using phenotypic tests [4–6]. Obtaining species affiliation by this method is rather expensive and, above all, time consuming, since sequences to be compared in reference databases are obtained through DNA extraction, and specific target amplification and sequencing. Thus, identification/classification of new isolates is not immediate.

In environmental or applied microbiology, prompt identification is not as important as in other fields (i.e. clinical/diagnostic microbiology) but the availability of a rapid identification tool is nevertheless widely sought-after.

Matrix-assisted laser desorption ionization–time of flight mass spectrometry (MALDI-TOF MS) is a high throughput reliable technology used to identify and analyse proteins. MALDI-TOF MS analysis of proteins from whole bacteria cells for identification purposes has long been demonstrated [7, 8], but its potential for bacterial routine identification has been assessed much later [9]. However, due to obvious social implications, most of the studies focused on microorganisms of clinical interest. MALDI-TOF MS had been already used to identify a wide array of microorganisms involved in bacterial diseases, including *Escherichia coli* and other members of the *Enterobacteriaceae* family [10], *Staphylococcus aureus* [11] and *Bacillus cereus* [12]. Nevertheless, the watershed in the use of this technology in microbial classification was the availability of commercial systems provided with robust databases and user-friendly software [9]. The first extensive study assessing the ability of MALDI-TOF MS to identify bacteria from clinical samples, carried out using dedicated software with an internal database, has been published in 2009 by Seng and co-workers [13]. This technique is rapidly acquiring increasing importance for fast microbial identification in human or animal clinical pathology, generally obtaining high concordance with other standard methods [14–18].

The use of MALDI-TOF MS for identification and classification of environmental bacteria has been definitely underrated. In addition, most of the available studies focused on single species or limited groups of microorganisms, while only a limited number investigated whole microbial communities [19–24]. In this context, the value of MALDI-TOF MS as an identification tool is still controversial and strongly dependent on the software/database used to analyse the results. Moreover, comparison with other credited methods is not always provided by literature, making the evaluation even more difficult. MALDI BioTyper (MB) is a rather new platform, developed by Bruker Daltonics, combining MALDI-TOF MS analysis with a dedicated database for bacterial identification. Even if it was designed for clinical uses, it has recently found applications in other branches of microbiology. However, its usefulness in environmental isolate identification is still scarcely estimated, especially in case of extreme environments where the unavailability of comprehensive reference spectra database further limits its use [25].

In this work, we evaluated the Bruker MALDI BioTyper as a fast and dependable method for the identification/classification of 45 bacterial strains previous isolated from an Arctic marine environment (Kandalaksha Bay, White Sea, Russia). The results were validated by comparison with the taxonomic identification obtained by 16S rDNA sequences analysis.

## Materials and methods

### Microorganisms and culture conditions

The bacterial strains used in this study were isolated in a previous work from sea water samples collected in Kandalaksha Bay (White Sea, Russia) and their 16S rDNA sequences were deposited in the NCBI GenBank database [26]. Isolation was followed by careful dereplication in order to eliminate the numerous duplicates of isolates obtained from single samples. Strains were maintained at 4°C in the culture collection of microorganisms of DEB (Department of Ecological and Biological Sciences, University of Tuscia) and sub-cultured when necessary on plate count agar (PCA) slants (Difco, USA).

Colonies for MALDI-TOF MS analysis where obtained from fresh cultures on PCA plates (incubated 24–48 h at 20°C). Media had been sterilised in autoclave for 20 min at 121°C, prior to sterilization pH had been adjusted to 7.0 ±0.2.

### Molecular phylogenetic analysis by 16S rDNA

The phylogenetic analysis, carried out in 2011 [26] and partially revised in 2014 [2], was repeated using the updated database, only for the 45 strains that have been submitted to MALDI BioTyper analysis. BLASTn was used to compare Kandalaksha Bay (KB) bacteria sequences to those of the NCBI nucleotide database https://blast.ncbi.nlm.nih.gov/Blast.cgi?PAGE_TYPE=BlastSearch) [27]. For attributions, only database sequences (listed in the BLAST report) having similarity ≥99% were considered. In most cases, due to uncertain species attribution from the Blast reports, identification was achieved by trees relating each KB bacteria sequence with those of type/reference strains and all other sequences that clustered undoubtedly together with the type/reference strains. MEGA 6.0 [28] was used for automatic 16S rDNA sequences alignment (by MUSCLE aligner) and Maximum Likelihood (ML) dendrogram construction.

For better clarity, a first phylogenetic tree is supplied to show relationships among all KB bacteria and their subdivision in classes. The attribution of KB strains was also showed by different trees related to *Pseudomonas*, *Serratia* and all the other analysed genera (*Others*). Bootstrap tests were conducted to infer the reliability of branch order, with 1000 pseudo-replicates.

### Sample preparation for MALDI-TOF mass spectrometry analysis

Both the direct colony (direct smear) and the extraction methods have been tested as reported by Alatoom et al. [29] with slight adjustments. For the direct colony method, loopfuls of bacteria from 24–48 h cultures were applied as thin films onto a 96-spot steel plate (Bruker Daltonics, Bremen, Germany) and dried at room temperature. Subsequently, 1 μl of MALDI matrix (a saturated solution of α-cyano-4-hydroxycinnamic acid (HCCA; Bruker Daltonics) in 50% acetonitrile and 2.5% trifluoroacetic acid) was applied onto the colony and allowed to dry before testing. For the extraction method, loopfuls of fresh cultures (as above) were suspended in 300 μl of molecular-grade water (Sigma-Aldrich, St. Louis, MO) and vortexed. Next, 900 μl of 100% ethanol (Sigma-Aldrich) was added, vortexed, and centrifuged (13000 rpm, 2 min). After discharge of supernatants, pellets were dried at room temperature, re-suspended in 50 μl of 70% formic acid (Fluka, Sigma-Aldrich, St.Louis, MO) and 50 μl of acetonitrile (Fluka), strongly mixed and centrifuged as above. Supernatants (1 μl) were spotted onto the plate and dried at room temperature before the addition of 1 μl of matrix. A Bacterial Test Standard (Bruker Daltonics) was included for each plate to calibrate the instrument and validate the run. For both methods, each sample was spotted at least five times on the plate.

### MALDI-TOF MS measurements and MALDI BioTyper identification

Mass spectra (2000–20000 Da) were automatically acquired, using the Autoflex III MALDI-TOF mass spectrometer equipped with a nitrogen laser, working in linear positive mode and controlled by the dedicated custom-made software FlexControl. Spectra were analysed using the Flex Analysis software (Bruker Daltonics, Bremen, Germany). The replicates showing intensity <10^4^ (arbitrary units), as well as those having a profile highly different from the others, were discarded. The MALDI BioTyper software, version 3.1 (Bruker Daltonics, Bremen, Germany) was used to process the raw spectra and to compare the spectra in order to classify the strains. The automation workflow of the MALDI BioTyper enables optimal sample acquisition (accumulation of typically 300 to 500 shots of high quality from different optimal spot positions), raw data processing and final identification in a few simple steps [29–30].

The standard Bruker interpretative criteria were applied as follows: unreliable identification (score 0.000–1.699); probable genus identification (score 1.700–1.999); secure genus and probable species identification (score 2.000–2.299); highly probable species identification (score 2.300–3.000) [31–32].

### Implementation of Maldi BioTyper database

MB database has been implemented with those entries (genera or species) that were not present, according to the following protocol:

One microliter of each bacterial extract was spotted eight times onto the steel plate and air-dried. Each sample was overlaid with 1 μL of the saturated matrix solution (see above) and air-dried. Each spot was measured three times. For each measurement, at least 1000 individual spectra (50 laser shots at 20 different spot positions) were accumulated and averaged. The resulting 24 spectra were carefully analysed and processed (i.e. smoothing, baseline subtraction, normalization, and peak picking) using the FlexAnalysis software and selected, to yield a minimum of 20 accurate spectra, based on their intensity. Spectra are then uploaded onto the MB database to create a single Main Spectrum (MSP) for each strain with the standard MALDI BioTyper MSP creation method (Bruker Daltonics, Bremen, Germany). A MSP contains the average mass and the average intensity of the selected peaks (representing most reproducible and typical for a certain bacterial strain) as well as the frequency of the peaks in multiple measurements.

## Results

### Molecular phylogenetic analysis by 16S rDNA

**Fig 1:**
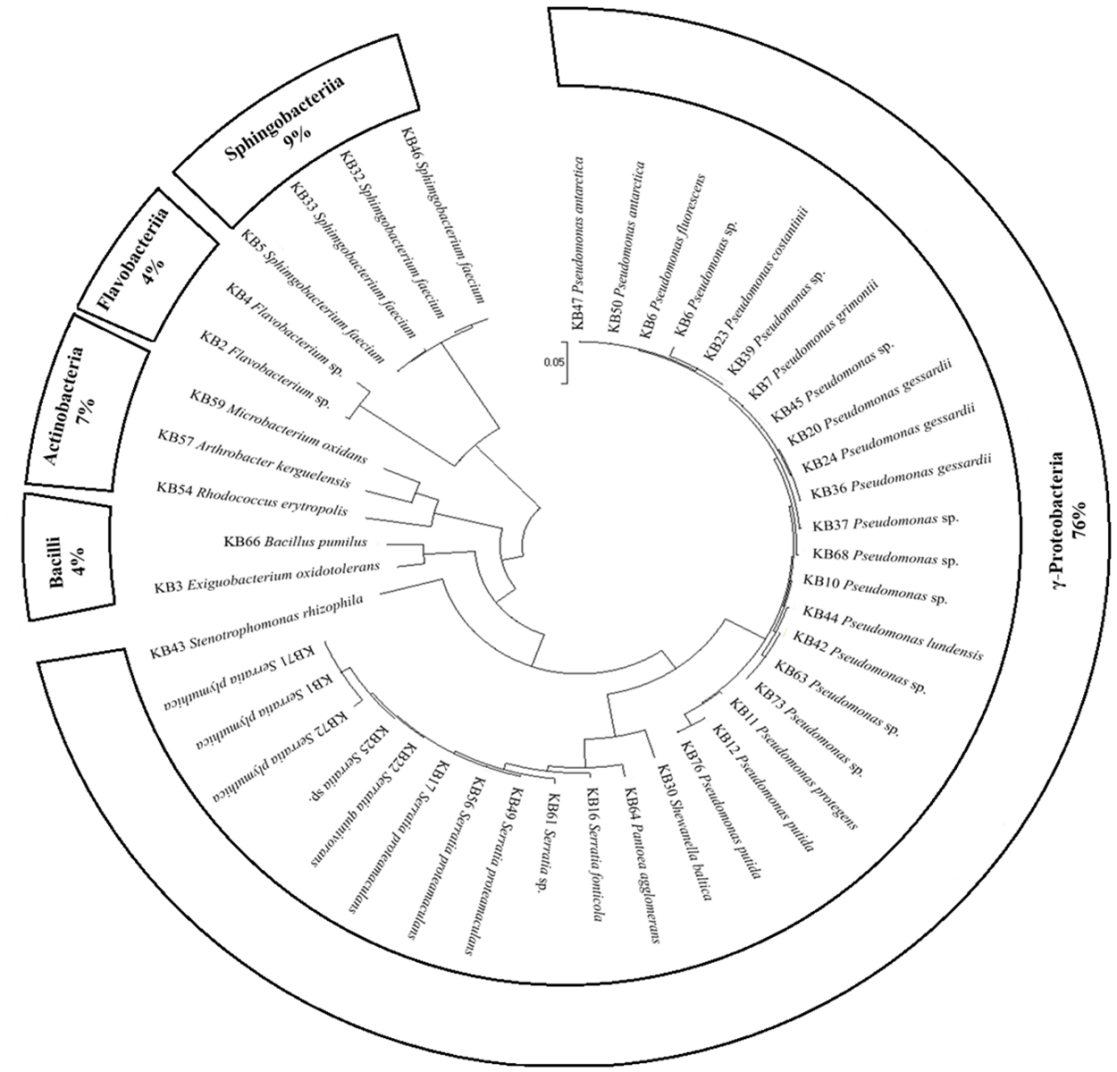
Maximum Likelihood phylogenetic dendrogram based on 16S rDNA sequences showing the relationship between all KB strains. Bar, 5 nt substitution per 100 nt.

**Fig 2:**
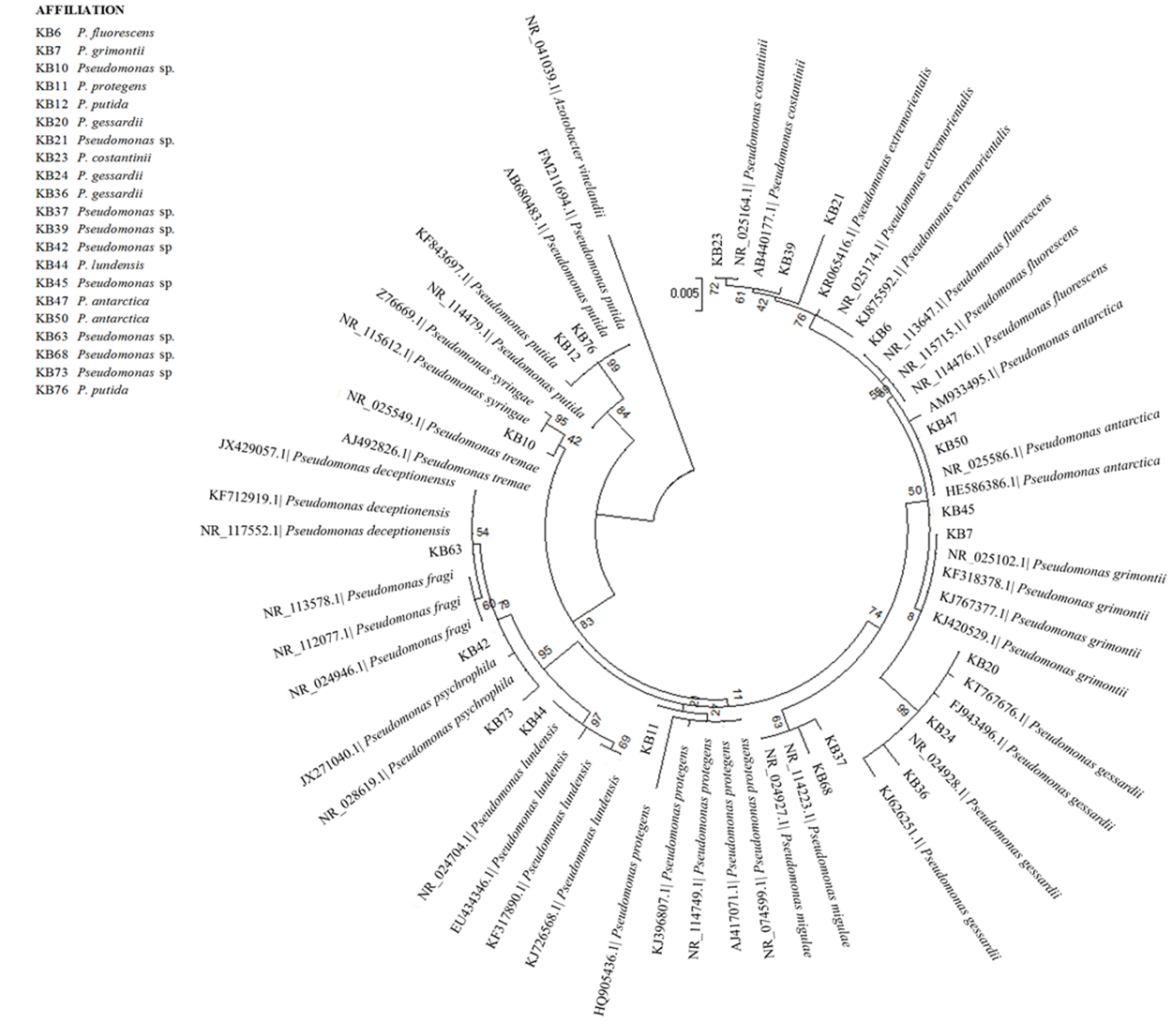
Maximum Likelihood phylogenetic dendrograms of KB strains belonged to *Pseudomonas* based on 16S rDNA sequences. Bootstrap values calculated for 1000 replications (values lower than 50 are not shown). Bar, 5 nt substitution per 1000 nt.

**Fig 3:**
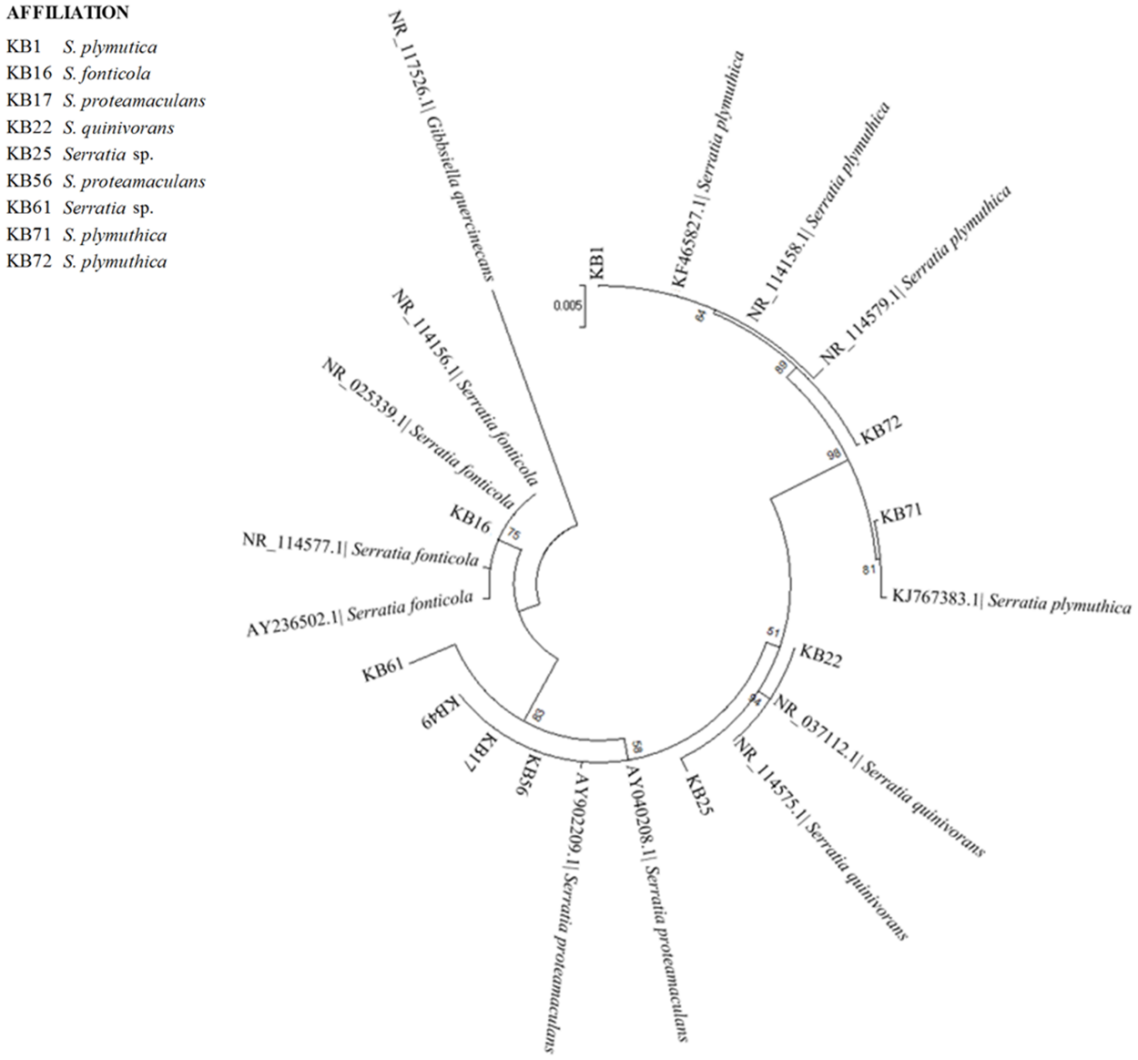
Maximum Likelihood phylogenetic dendrograms of KB strains belonged to *Serratia* based on 16S rDNA sequences. Bootstrap values calculated for 1000 replications (values lower than 50 are not shown). Bar, 5 nt substitution per 1000 nt.

**Fig 4:**
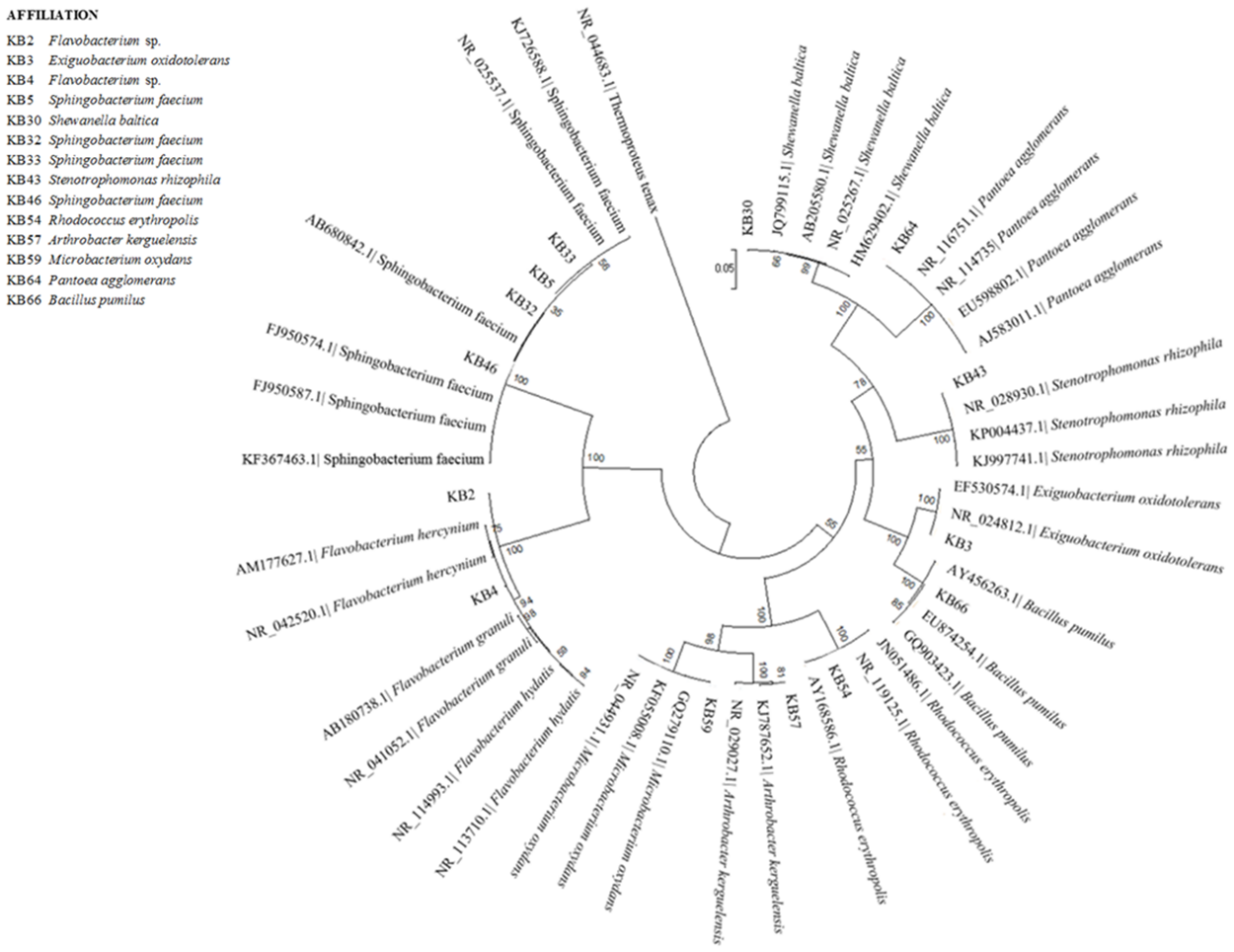
Maximum Likelihood phylogenetic dendrograms of KB strains belonged to the “Other” group, based on 16S rDNA sequences. Bootstrap values calculated for 1000 replications (values lower than 50 are not shown). Bar, 5 nt substitution per 100 nt.

The dendrogram reported in Fig 1 showed the phylogenetic relationships among all KB bacteria. The tree is clearly clustered according to 5 different classes: γ;-Proteobacteria, Bacilli, Actinobacteria, Flavobacteriia and Sphingobacteriia. *Pseudomonas* and *Serratia* (γ-Proteobacteria) were the principal genera with 21 and 10 strains, respectively. Various other minority genera (*Arthrobacter*, *Bacillus*, *Exiguobacterium*, *Flavobacterium*, *Microbacterium*, *Pantoea*, *Rhodococcus*, *Shewanella*, *Sphingobacterium*, *Stenotrophomonas*,) were present with few strains each. Thus, for simplicity, the KB bacteria could be divided in three main groups: *Pseudomonas*, *Serratia* and “*Other*” (gathering the remaining genera). For better comprehension, their phylogenetic relationships have been organised in three different ML dendrograms, resuming the information obtained for each strain by the specific tree (Figs 2–4).

As for *Pseudomonas*, attribution at the species level was obtained for 12 strains out of 21 (Fig 2): KB11, clustering outside from the *P*. *protegens* group, was clearly attributed to this species thanks to the single ML tree. KB20, KB24 and KB36 belonged to *P*. *gessardii*; KB12 and KB76 to *P*. *putida*; KB47 and KB50 to *P*. *antarctica*; KB6 to *P*. *fluorescens*; KB7 to *P*. *grimontii*; KB23 to *P*. *costantinii*; KB44 to *P*. *lundensis*. For KB10, KB21, KB37, KB39, KB42, KB45, KB63, KB68 and KB73, attribution was possible at the genus level only (*Pseudomonas* sp.).

Fig 3 reports the phylogenetic relationships among *Serratia* strains. Clustering permitted good species attribution for the majority of the strains (7 out of 10): KB1, KB71 and KB72 belonged to *S*. *plymuthica*; KB17, KB49, KB56, to *S*. *proteamaculans* and KB16 to *S*. *fonticola*, respectively. For strains KB25 and KB61 attribution to the species level was not possible.

Relationships among the *Other* strains are reported in Fig 4, which is clearly clustered according to the various genera. Attribution at the species level was obtained for most of the strains (12 out of 14): KB3 belonged to *Exiguobacterium oxidotolerans*; KB5, KB32, KB33 and KB46 to *Sphingobacterium faecium*; KB30 to *Shewanella baltica*; KB43 to *Stenotrophomonas rhizophila*; KB54 to *Rhodococcus erythropolis*; KB57 to *Arthrobacter kerguelensis*, KB59 to *Microbacterium oxydans*; KB64 to *Pantoea agglomerans*; KB66 to *Bacillus pumilus*; KB2 and KB4 to *Flavobacterium* sp.

For some strains, the attribution obtained by present revision differed from those carried out in 2011 [26] and 2014 [2]. Most of the reattributions regarded strains belonging to *Pseudomonas*. In particular, KB11, KB20, KB24 and KB36, previously identified as *P*. *fluorescens*, are currently attributed to *P*. *protegens* or *P*. *gessardii*. Strains KB32 and KB46 (*Sphingobacterium* sp.), previously identified at the genus level only, are currently attributed to *S*. *faecium*. Attribution of strain KB61 in 2011 was only at the genus level (*Serratia* sp.), while in 2014 it was updated to *S*. *subantarctica*. Since the *S*. *subantarctica* sequence has been removed from the database, current attribution returned to *Serratia* sp.

### Identification by MALDI BioTyper and comparison with 16S rDNA

The preliminary tests, carried out to choose the right spotting procedure, indicated that only very low scores (<1.900) were obtained by the direct smear method; thus, the extraction method was used for all the samples.

Table 1 shows the attributions obtained by MB analysis for KB strains. All the bacteria have been identified at least at the genus level with the exception of KB3 (identified as *Exiguobacterium oxidotolerans* by 16S rDNA) for which no reliable identification has been obtained. However, since the genus was not present in the MB database, identification concordance at the genus level between MB and 16S rDNA analyses could be considered as 100%. Concordance has been calculated considering the results from 16S rDNA analysis (gold standard) as a reference.

**Table 1:** KB strains attributions obtained by MB and concordance (at the species level) with the 16S rDNA analysis.

STRAIN	ACC. N°	IDENTIFICATION	SCORE	CONC
KB 1	JF327440	*Serratia plymuthica*	2.318	+
KB 2	JF327441	*Flavobacterium* sp.	1.958	+
KB 3	JF327442	Not reliable identification	/	/
KB 4	JF327443	*Flavobacterium hydatis*	2.216	-
KB 5	JF327444	*Sphingobacterium faecium*	2.415	+
KB 6	JF327445	*Pseudomonas fluorescens*	2.316	+
KB 7	JF327446	*Pseudomonas marginalis*	2.333	-
KB 10	JF327447	*Pseudomonas chlororaphis*	2.149	-
KB 11	JF327448	*Pseudomonas chlororaphis*	2.136	-
KB 12	JF327449	*Pseudomonas* sp.	1.723	-
KB 16	JF327450	*Serratia fonticola*	2.119	+
KB 17	JF327451	*Serratia proteamaculans*	2.376	+
KB 20	JF327452	*Pseudomonas gessardii*	2.157	+
KB 21	JF327453	*Pseudomonas gessardii*	2.255	-
KB 22	JF327454	*Serratia proteamaculans*	2.432	-
KB 23	JF327455	*Pseudomonas antarctica*	2.181	-
KB 24	JF327456	*Pseudomonas gessardii*	2.170	+
KB 25	JF327457	*Serratia proteamaculans*	2.476	-
KB 30	JF327458	*Shewanella baltica*	2.206	+
KB 32	JF327460	*Sphingobacterium faecium*	2.236	+
KB 33	JF327461	*Sphingobacterium faecium*	2.371	+
KB 36	JF327462	*Pseudomonas gessardii*	2.216	+
KB 37	JF327463	*Pseudomonas gessardii*	2.159	-
KB 39	JF327465	*Pseudomonas fluorescens*	2.153	-
KB 42	JF327467	*Pseudomonas taetrolens*	2.055	-
KB 43	JF327468	*Stenotrophomonas rhizophila*	2.466	+
KB 44	JF327469	*Pseudomonas fragi*	2.243	-
KB 45	JF327470	*Pseudomonas extremorientalis*	2.164	-
KB 46	JF327471	*Sphingobacterium* sp.	1.808	-
KB 47	JF327472	*Pseudomonas antarctica*	2.530	+
KB 49	JF327473	*Serratia proteamaculans*	2.432	+
KB 50	JF327474	*Pseudomonas antarctica*	2.338	+
KB 54	JF327477	*Rhodococcus* sp.	1.777	-
KB 56	JF327478	*Serratia proteamaculans*	2.278	+
KB 57	JF327479	*Arthrobacter kerguelensis*	2.166	+
KB 59	JF327481	*Microbacterium maritypicum*	2.111	-
KB 61	JF327482	*Serratia liquefaciens*	2.326	-
KB 63	JF327483	*Pseudomonas taetrolens*	2.299	-
KB 64	JF327484	*Pantoea agglomearns*	2.354	+
KB 66	JF327485	*Bacillus simplex*	2.218	-
KB 68	JF327486	*Pseudomonas koreensis*	2.130	-
KB 71	JF327487	*Serratia plymuthica*	2.461	+
KB 72	JF327488	*Serratia plymuthica*	2.414	+
KB 73	JF327489	*Pseudomonas taetrolens*	2.196	-
KB 76	JF327491	*Pseudomonas* sp.	1.802	-

ACC. N° = GenBank accession number; SCORE = identification score given by MB (unreliable identification: 0.000–1.699; probable genus identification: 1.700–1.999; secure genus and probable species identification: 2.000–2.299; highly probable species identification: 2.300–3.000); CONC = attribution concordance between MB and 16S rDNA analyses at the species level.

According to MB directions, the identification at the species level could be considered as highly probable (score 2.300–3.000) and probable (score 2.000–2.299) for 16 and 23 strains, respectively. For the remaining five strains, attribution was only at the genus level (score 1.700–1.999).

At the species level, the overall concordance between the two methods was ca 48% (Strain KB3 is not included) while, considering the various groups, it was ca 29, 70 and 62% for *Pseudomonas*, *Serratia* and *Others*, respectively.

### Implementation of MB database

**Fig 5:**
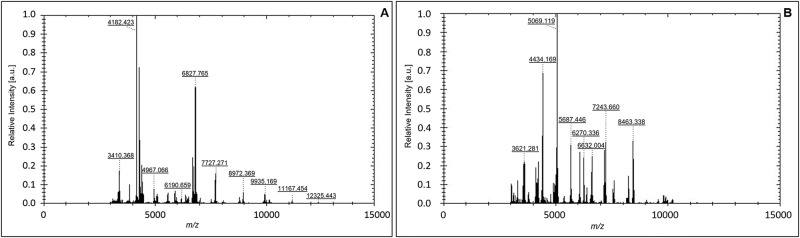
Main spectra of the species *Exiguobacterium oxidotolerans* (a) and *Pseudomonas costantinii* (b) obtained by MALDI-TOF analysis. The relative intensity of the ions (arbitrary units, a.u.) and their mass to charge ratio (*m/z*) are shown on the *y* and *x-axis*, respectively.

The genus *Exiguobacterium* and the species *Pseudomonas costantinii* were not present in the MB database. Strains KB3 and KB23, were classified by 16S rDNA analysis as *Exiguobacterium oxidotolerans* and *Pseudomonas costantinii*, respectively. Since their attribution was quite satisfying, the corresponding MSP spectra (Fig 5) have been added to the MB database (homemade implementation), according to the procedure reported in M&M.

## Discussion

### Molecular phylogenetic analysis by 16S rDNA

The phylogenetic analysis was obtained according to the up-to-date “nucleotide collection” database of NCBI GenBank. In most cases, the Blast report did not supply the univocal result necessary for direct species attribution. Thus, for each strain, attribution was obtained by single phylogenetic ML trees, using only sequences from type/reference strains and all other sequences that clustered undoubtedly together with them (figures not shown).

For various strains, affiliation was different from that obtained previously [2, 26]. This is not surprising: GenBank is an open database and free submission of DNA sequences leads to its continuous integration with new entries and removal/revision of misidentified records. Free access to the database is an advantage since it provides large and comprehensive information, but on the other hand, it affects data quality and reliability control. Actually, there is strong evidence of mistakes within the database and the phylogenetic analysis should be carried out using reference or type strains as the principal benchmarks.

In our case, most of the re-attributions involved strains of *Pseudomonas*, which is known as one of the most heterogeneous and biodiverse genera. Its taxonomy is very problematic and has undergone many changes. As demonstrated, the sole use of the 16S rDNA target could be insufficient for its low resolution at the intrageneric level. This marker, however, is often included in the Multilocus Sequence Analysis (MLSA), which provides an accurate approach for the phylogeny of this genus [33–37]. However, the attribution obtained by 16S rDNA for some of our strains appeared rather convincing and it has been employed to infer the phylogeny.

### Identification by MALDI BioTyper and comparison with 16S rDNA analysis

MB is principally employed when a fast identification is required (i.e. clinical microbiology). For this reason, most authors concluded that the direct smear method was preferable due to the much shorter preparation time, since the marginally higher identification scores obtained with the extraction protocol would not justify the extra preparation time [38–41]. In our case, score differences between the two methods were not marginal: as said, with direct smear, most of them were too low (<1.900) for the identification at the species level. Moreover, the extraction procedure is not particularly time-consuming, requiring less than one hour for 45 strains. In addition, if appropriately stored, the extracts are stable, allowing subsequent tests with no need for further cell cultivation [42–43].

Identification by 16S rDNA analysis is widely considered the gold standard. MB could be an interesting alternative, particularly in certain fields where fast analysis is required, such as human and animal clinical microbiology. In these areas, various papers validated the emerging role of MB in the rapid and reliable routine identification of pathogens. High concordance with other standard phenotypic/genotypic methods has been shown, even if most of the work regarded specific microbial groups only [14–18, 44–45]. For example, according to the study on animal pathogens by Hijazin and co-workers [15], MB discriminating power was comparable to that shown by various DNA targets analyses. These authors obtained 100% of concordance between the attributions by MB and those by previous genotypic methods. Rapid and reliable identification of *Arcobacter*, *Helicobacter* and *Campylobacter* species had been obtained by MB analysis in the work of Alispahic et al. [14], showing 98% of concordance with the 16S rDNA PCR-RFLP method and solving the issue arisen by phenotypical similarities among species of these genera. Sogawa and co-workers [17] investigated 468 strains of clinical interest, obtaining ca 92% of concordance between MB and other standard methods including 16S rDNA analysis.

As said, in environmental microbiology, the use of MALDI-TOF MS for identification and classification purposes has been underrated and the majority of the investigations dealt with single species or selected microbial groups. Extensive studies on total bacterial environmental communities are limited in number and sometime other mass spectrometry systems were used [19–24]. In general, in contrast with the results obtained in clinical investigations, concordance between MB and 16S rDNA analyses was rather low. Oberbeckmann and co-workers [46], studying 44 environmental *Vibrio* isolates from temperate waters, compared MB and 16S rDNA/rpoB sequence analyses obtaining 43% of concordance. Uhlik et al. [23] isolated 54 cultured biphenyl-metabolizing bacteria from contaminated soil and obtained high concordance between MB and 16S rDNA analysis at the genus level only. Even if Mulec et al. [47] did not compare MB identification with other methods, their work indicated that only 40% of the bacterial strains isolated from a thermal mud were identified by MB.

Thus, together with low concordance with other standard methods, MB would have a general low identification capability of environmental strains. This low concordance is principally due to the MB database design: mainly developed for clinical uses and scarcely performant in relation to very specific microbial groups, including those isolated from peculiar environments.

The results of our work showed that, considering all the strains analysed, concordance was slightly higher (48%) than that reported in literature. However, it is worth noting that big differences were recorded among the various groups and, for the *Serratia* and *Other* strains, concordance was quite high (70 and 62%, respectively). By contrast, for *Pseudomonas* it was very low (29%), somehow confirming the problematic identification and phylogeny of this genus, as discussed above.

On the whole, if we consider the average results reported by other authors for environmental bacteria, our figures resulted quite satisfying. Nevertheless, various cited works used obsolete databases; concordance calculated using the newest releases would probably be higher.

However, it is evident that there is still a consistent lack of information regarding the real value of MB as an identification tool for environmental bacteria and more studies are necessary, together with substantial improvements of its database. In our study, we implemented the MB database “homemade”, adding the genus *Exiguobacterium* (with the species *E*. *oxidotolerans*) and the species *Pseudomonas costantinii*.

### Implementation of MB database

Most of the problems found in phylogenetic analysis are related to the GenBank database that, as discussed previously, is an open source with limited control over the entries uploaded by the users; only reference sequences are carefully verified. In addition, clearly wrong entries, affecting the phylogenetic analysis, can be removed only by the depositors. By contrast, MB database is closed and mainly build using type strains permitting the access to very coherent controlled data. However, this feature significantly reduces the flexibility required to cope with the extremely fast discovery of new organisms. The low concordance scores, often found in literature (see above), could be often imputable to lack of entries for specific environmental microbial genera or species. Although the database used in this study is a more recent version compared with those used by other authors, we can confirm that there is a continuous need of spectra database expansion to improve the identification ability of MALDI TOF MS for environmental bacteria [25, 48–49]. However, MB gives the possibility for “homemade” database implementation adding the spectra corresponding to missing genera or species.

In our case, the following isolates have been added to the MB database according to the procedure reported in Materials and Methods: *Exiguobacterium oxidotolerans* (KB3) and *Pseudomonas costantinii* (KB23). It is worth nothing that genus *Exiguobacterium* was not present. The corresponding MSP spectra are reported in Fig 5.

## Conclusions

On the whole, the results of this study, showing quite high concordance between the identifications obtained by MB and 16S rDNA analyses, suggested the applicability of the MB platform as a fast and powerful tool for low-cost screening, also for bacteria isolated from extreme environments. Even though the analysis of the ribosomal gene is still important to identify new isolates for preliminary and/or routine analysis, MB can be chosen for its easy and rapid sample preparation. However, for a more accurate identification, MB database needs to be implemented with a higher number of environmental strains.
